# Retrospective analysis of the effect of SGLT-2 inhibitors on renal function in patients with type 2 diabetes in the real world

**DOI:** 10.3389/fphar.2024.1376850

**Published:** 2024-08-05

**Authors:** Rongjing Song, Qiaoyu Hou, Xiuying Zhang, Wei Zhao, Gang Liu, Meng Li, Xiaohong Zhang, Linong Ji

**Affiliations:** ^1^ Department of Pharmacy, Peking University People’s Hospital, Beijing, China; ^2^ Department of Clinical Pharmacy, Qilu Hospital of Shandong University Dezhou Hospital, Dezhou, China; ^3^ Department of Endocrinology and Metabolism, Peking University People’s Hospital, Peking University Diabetes Centre, Beijing, China; ^4^ Department of Obstetrics and Gynecology, Peking University People’s Hospital, Beijing, China

**Keywords:** sodium-glucose cotransporter-2 inhibitors, type 2 diabetes mellitus, renal function, estimated glomerular filtration rate, urinary albumin/creatinine ratio

## Abstract

**Introduction:**

The protective effect of sodium–glucose cotransporter-2 (SGLT-2) inhibitors on the kidneys has been widely recognized. However, limited research has reported the changes in estimated glomerular filtration rate (eGFR) of real-world patients with type 2 diabetes mellitus (T2DM) over time after administration of SGLT-2 inhibitors. This study aimed to reflect the trend of eGFR changes over time in T2DM patients having different baseline eGFR after SGLT-2 inhibitors administration in the real world.

**Methods:**

A single-center retrospective study was performed in a tertiary public hospital in Beijing, China. In total, 998 outpatients with T2DM who initiated SGLT-2 inhibitors treatment were included in the study. The changes in eGFR, urinary albumin/creatinine ratio (UACR), and glycolipid metabolism indicators were analyzed during the 18-month follow-up period.

**Results:**

The eGFR levels significantly decreased to their lowest point (−3.04 mL/min/1.73 m^2^) in the first 3 months after initiation of SGLT-2 inhibitors treatment, however, gradually returned to the baseline level after 1 year. Compared to the subgroup with eGFR >90 mL/min/1.73 m^2^, improvements in renal function were more significant in patients with T2DM from the 60 < eGFR ≤90 mL/min/1.73 m^2^ and eGFR ≤60 mL/min/1.73 m^2^ subgroups after treatment with SGLT-2 inhibitors. Similarly, SGLT-2 inhibitors reduced the UACR in patients with diabetic nephropathy.

**Conclusion:**

This study further confirmed the real-world long-term protective effect of SGLT-2 inhibitors on the kidneys of patients with T2DM, which is not related to baseline renal function and blood glucose.

## Introduction

Type 2 diabetes mellitus (T2DM) is one of the most common factors leading to renal failure and lacks effective prevention and treatment methods (Saran et al., 2017; Warren et al., 2018). The number of patients receiving renal replacement therapy for renal failure is reported to exceed 5 million worldwide in 2030 (Liyanage et al., 2015). Therefore, effective measures to improve the renal prognosis of patients with T2DM have become an area of great concern. In recent years, sodium–glucose cotransporter-2 (SGLT-2) inhibitors, a new class of oral hypoglycemic drugs that include canagliflozin, empagliflozin, and dapagliflozin, have shown renal benefits independent of hypoglycemic effects both in outcomes research and in clinical practice (Vlado et al., 2018; Heerspink et al., 2020a; De Nicola et al., 2020; Jongs et al., 2021). These improvements mainly include reducing proteinuria and delaying the deterioration of renal function (Ma et al., 2023). The CREDENCE trial, the first clinical trial to report the impact of SGLT-2 inhibitors on the kidneys, showed that the risk of renal failure was lower in the canagliflozin group than in the placebo group among patients with T2DM combining chronic kidney disease (CKD) (Perkovic et al., 2019). The subsequent EMPEROR trial showed that empagliflozin slowed the rate of renal function decline both in patients with and without CKD regardless of the severity of renal injury at baseline (Zannad et al., 2021). A large phase III clinical study, DAPA-CKD, showed that dapagliflozin treatment could delay the progression of kidney disease and reduce the risk of the main endpoint (composite of a sustained decline in the estimated glomerular filtration rate (eGFR) of at least 50%, end-stage kidney disease, or death from renal or cardiovascular causes) by 5.3%, including in patients without diabetes (Heerspink et al., 2020a). Thus, based on such a large number of clinical trial results, multiple countries have successively approved SGLT-2 inhibitors for the treatment of CKD (Herrington et al., 2021; Administration USFD Home-News, 2024; Agency, 2024; Astrazeneca, 2024).

Multiple mechanisms have been reported to be involved in the protective effect of SGLT-2 inhibitors on the kidneys (Heerspink et al., 2016). eGFR usually increases with the development of diabetes, which is an indicator of the development of diabetic nephropathy. SGLT-2 inhibitors reduce glucose levels by inhibiting the re-absorption of glucose and sodium in the proximal tubules, increasing their transport to the distal renal tubules, normalizing tubular bead feedback signals, and alleviating over filtration (Vallon and Thomson, 2020). The increase in glomerular perfusion leads to an increase in vasoconstriction of afferent arterioles, followed by a decrease in glomerular perfusion and intraglomerular pressure. These changes rapidly reduce the eGFR, which, however, gradually stabilizes over time (Wanner et al., 2016). This phenomenon was confirmed by the CREDENCE and DAPA-CKD trials (Perkovic et al., 2019; Heerspink et al., 2020a). Moreover, Zannad et al. found that the eGFR may decrease slightly following SGLT-2 inhibitors administration, which does not affect renal function (Zannad et al., 2022).

However, the research included in these clinical trials is highly population selective, which cannot directly reflect the real-world trend of the renal function change over time in patients with T2DM after SGLT-2 inhibitors administration. Limited studies have reported the impact of SGLT-2 inhibitors on eGFR in the real world population, (Fadini et al., 2018; Kosiborod et al., 2018; Udell et al., 2018; Fadini et al., 2019; Kinguchi et al., 2019; Lin et al., 2019; Sezai et al., 2019; Heerspink et al., 2020b; Pasternak et al., 2020), with only a few having reported specific renal test results, such as eGFR and urinary albumin/creatinine ratio (UACR) (Heerspink et al., 2020b; Lin et al., 2021). Therefore, this study aims to conduct a retrospective analysis of the real-world data of outpatients with T2DM using SGLT-2 inhibitors with different baseline renal functions in mainland China. It is expected that this study will extend the existing evidence from the clinical trials to a wider and more heterogeneous population to provide further support, and a well-informed rationale behind the clinical application of SGLT-2 inhibitors, while ensuring that patients use these more safely and effectively.

## Methods

### Data sources and patient selection

This was a single-center retrospective retrospective study. The medical records of 4,734 outpatients treated with SGLT-2 inhibitors from January 2020 to June 2021 were obtained from the clinical data center of Peking University People’s Hospital, Beijing China. Three SGLT-2 inhibitors, canagliflozin, empagliflozin, and dapagliflozin, were available in our hospital during the study period. Patients who met the following conditions were included in the study: 1) the first administration of SGLT-2 inhibitors in a patient was only after a corresponding prescription issued by our hospital; 2) prescriptions containing SGLT-2 inhibitors were issued at least three times during the study period; 3) patients had relevant test results, including those for eGFR, at or within 9 months prior to the first use of SGLT-2 inhibitors (baseline period was from 9 months before medication to the day of prescription); and 4) patients who had at least one relevant laboratory test result, including that for eGFR, within 9 months after the first prescription of SGLT-2 inhibitors. Cases with any of the following conditions were excluded: 1) < 18 years old; 2) patients with type 1 diabetes or 3) gestational diabetes; 4) patients with cancer; and 5) patients who failed to meet the inclusion criteria. These inclusion and exclusion criteria were used to construct the patient database for this research. The study design was approved by the Ethics Committee of Peking University People’s Hospital, and all study participants provided informed consent.

### Data extraction

Baseline data included laboratory results extracted at 1, 3, 6, 9, 12, 15, and 18 months after the first prescription of SGLT-2 inhibitors. First, the following information was collected at baseline: age, sex, body mass index (BMI), diabetes diagnosis, course, and complications (including diabetic retinopathy, nephropathy, and peripheral neuropathy), category of SGLT-2 inhibitors, department where SGLT-2 inhibitors were first prescribed, other comorbidities (including cerebrovascular disease, coronary heart disease, heart failure, hypertension, hyperlipidemia, and hyperuricemia), and laboratory examination data, including reports for eGFR, blood uric acid (UA), triglyceride (TG), total cholesterol (TC), low-density lipoprotein cholesterol (LDL-C), high-density lipoprotein cholesterol (HDL-C), UACR, and glycated hemoglobin A1c (HbA1c).

Second, the following data of patients during an observation period of 18 months after the first prescription of SGLT-2 inhibitors was collected: 1) the prescription status of SGLT2i during the observation period; 2) concurrent usage of other hypoglycemic medications, including metformin, sulfonylurea, glinides, acarbose, thiazolidinedione, dipeptidyl peptidase-4 inhibitors, glucagon-like peptide-1 receptor agonists, and insulin, and of other drugs for chronic diseases, including angiotensin-converting enzyme (ACE) inhibitors, angiotensin II receptor blocker (ARB), calcium channel blocker (CCB), antihypertensive drugs, β-receptor blockers, diuretics, statins, antiplatelet drugs such as aspirin, and urate-lowering therapy drugs; and 3) the outcomes of laboratory examinations, for the same parameters as mentioned for baseline measurements, at 1, 3, 6, 9, 12, 15, and 18 months after the first prescription of SGLT-2 inhibitors.

### Statistical analyses

SPSS22.0 software was used to conduct statistical analyses of the collected data. When the sample size was ≤50, the Shapiro–Wilk test was used to determine whether the measured data conformed to a normal distribution; when the sample size was >50, the Kolmogorov–Smirnov test was used. The variables of a normal distribution are expressed as (mean ± standard error of the mean), and the variables of a non-normal distribution were expressed as median and interquartile range. The counting data is expressed as n (%). In univariate analysis, if the difference between two groups of measured data conformed to a normal distribution, a paired sample *t*-test was used for comparison between the two groups, and the Pearson correlation coefficient was used for correlation analysis of continuous variables. If the difference between the two groups of measured data was not normally distributed, the Wilcoxon rank-sum test was used for comparison between the two groups, and the Spearman correlation coefficient was used for correlation analysis. GraphPad Prism software was used for drawing. P values <0.05 were considered to be statistically significant.

The study focused on the trend of the patient laboratory indicators, especially eGFR changes over time within 18 months after SGLT-2 inhibitors treatment in patients, as well as the cutoff point of the gradual increase in eGFR after SGLT-2 inhibitors treatment. We performed a stratified analysis based on baseline eGFR levels. According to the baseline eGFR value, the included cases were divided into three subgroups: eGFR >90 mL/min/1.73 m^2^, 60 < eGFR ≤90 mL/min/1.73 m^2^, and eGFR ≤60 mL/min/1.73 m^2^. In addition to the overall analysis, other laboratory indicators, such as UACR, HbA1c, and UA were also divided into 2–3 different subgroups according to the normal range of laboratory tests.

## Results

### Study population characteristics

**FIGURE 1:**
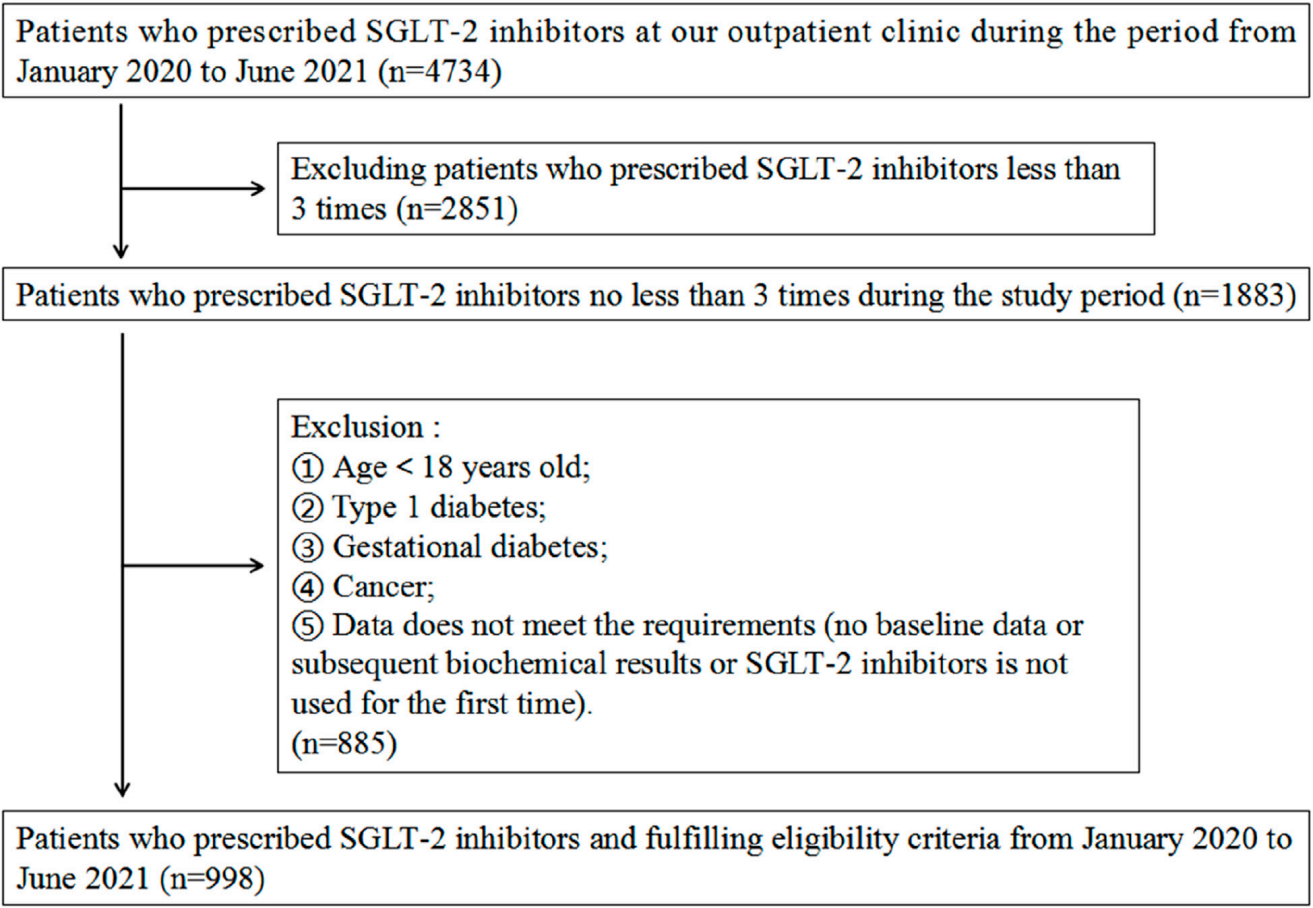
Flowchart of the patients’ screening process for this study.

A total of 4,734 patients using SGLT-2 inhibitors were identified in the outpatient department. The specific case screening details are illustrated in Figure 1. After screening according to the inclusion and exclusion criteria, 998 patients were included in this study, the population characteristics are shown in Table 1. Among the 998 patients, 656 were males (65.7%) and 342 were females (34.3%), with an average age of 60.09 ± 12.13 years. The average BMI was 26.80 ± 3.93 kg/m^2^. All patients had confirmed diagnoses of T2DM, among them 343 patients had diabetes for <5 years, 164 for 5–10 years, 349 for 10–20 years, and 142 for >20 years. Most patients had developed diabetes-related complications, such as diabetes peripheral neuropathy (35.8%), nephropathy (29.4%), and retinopathy (16.8%). The average HbA1c level of all 998 patients was 8.27% ± 1.66%, and eGFR was 87.19 ± 20.57 mL/min/1.73 m^2^ at baseline. The proportions of patients with concomitant chronic diseases were high: 816 (81.8%) suffered from hypertension, 757 (75.9%) from hyperlipidemia, 498 (49.9%) from coronary heart disease, 388 (38.9%) from cerebrovascular disease, and 261 (26.2%) from heart failure. The departments that prescribed SGLT-2 inhibitors mainly included endocrinology (78.4%), nephrology (12.3%), cardiology (4.7%), geriatrics (2.5%), and other departments (2.1%). The specific concomitant medications are shown in Supplementary Table S1. Among the 998 patients, 69 patients were initially treated with SGLT-2 inhibitors monotherapy, while most patients were treated with combination therapy based on SGLT-2 inhibitors: metformin (14.1%), metformin and other hypoglycemic drugs (63.2%), and hypoglycemic drugs other than metformin (15.7%). The combined use of medications for other chronic diseases included ACEI (8.0%), ARB (46.2%), β receptor blockers (36.6%), CCB (31.3%), diuretics (3.3%), statins (71.1%), antiplatelet drugs (19.3%), and urate-lowering therapy drugs (8.2%).

**TABLE 1:** Baseline characteristics of 998 patients including overall and subgroups stratifed by baseline eGFR levels (eGFR ≤60, 60–90, >90 mL/min/1.73 m^2^).

Characteristics	Overall	eGFR≤60 mL/min/1.73m^2^	60<eGFR≤90 mL/min/1.73m^2^	eGFR>90 mL/min/1.73m^2^
	N = 998	N = 117	N = 362	N = 519
Male, n (%)	656 (65.7)	69 (59.0)	261 (72.1)	326 (62.8)
Age (years), mean (SD)	60.09 (12.13)	67.31 (11.93)	65.26 (10.52)	54.84 (10.75)
BMI (kg/m^2^), mean (SD)	26.80 (3.93)	25.73 (3.84)	26.61 (3.77)	27.09 (4.00)
Diagnosis of diabetes, n (%)	998 (100)	117 (100)	362 (100)	519 (100)
Duration of diabetes (years), n (%)				
≤5	343 (34.4)	33 (28.2)	112 (30.9)	198 (38.2)
5–10	164 (16.4)	21 (17.9)	45 (12.4)	98 (18.9)
10–20	349 (35.0)	36 (30.8)	139 (38.4)	174 (33.5)
≥20	142 (14.2)	27 (23.1)	66 (18.2)	49 (9.4)
Diabetic complications, n (%)				
None	493 (49.4)	34 (29.1)	174 (48.1)	285 (54.9)
Diabetic retinopathy	168 (16.8)	36 (30.8)	61 (16.9)	71 (13.7)
Diabetic nephropathy	294 (29.4)	68 (58.1)	123 (34.0)	103 (19.8)
Diabetic peripheral neuropathy	357 (35.8)	51 (43.6)	133 (36.7)	173 (33.3)
Baseline HbA1c (%), mean (SD)	8.27 (1.66)	7.91 (1.70)	8.03 (1.55)	8.49 (1.69)
History of cardiovascular disease, n (%)				
Coronary heart disease	498 (49.9)	72 (61.5)	218 (60.2)	208 (40.1)
Heart failure	261 (26.2)	45 (38.5)	126 (34.8)	90 (17.3)
Hypertension	816 (81.8)	115 (98.3)	326 (90.1)	375 (72.2)
Hyperlipidemia	757 (75.9)	90 (76.9)	297 (82.0)	370 (71.3)
Cerebrovascular disease	388 (38.9)	67 (57.3)	161 (44.5)	160 (30.8)
SGLT-2 inhibitors, n (%)				
Empagliflozin	504 (50.5)	49 (41.9)	184 (50.8)	271 (52.2)
Dapagliflozin	270 (27.1)	30 (25.6)	94 (26.0)	146 (28.1)
Canagliflozin	193 (19.3)	29 (24.8)	77 (21.3)	87 (16.8)
Others^a^	31 (3.1)	9 (7.7)	7 (1.9)	15 (2.9)
Department SGLT-2 inhibitors first prescribed, n (%)				
Endocrinology department	782 (78.4)	64 (54.7)	264 (72.9)	454 (87.5)
Nephrology department	123 (12.3)	43 (36.8)	50 (13.8)	30 (5.8)
Cardiology department	47 (4.7)	7 (6.0)	21 (5.8)	19 (3.7)
Geriatrics department	25 (2.5)	2 (1.7)	15 (4.1)	8 (1.5)
Other departments	21 (2.1)	1 (0.8)	12 (3.3)	8 (1.5)
Baseline eGFR (ml/min/1.73m^2^), mean (SD)	87.19 (20.57)	47.57 (9.39)	70.75 (8.22)	102.72 (9.46)

Abbreviations are as follows: eGFR, estimated glomerular fltration rate; SD, standard deviation; BMI, body mass index; HbA1c, glycated hemoglobin A1c; SGLT-2, inhibitors, Sodium glucose cotransporter-2 inhibitors.

Others a: Using multiple SGLT-2 inhibitors.

### Analysis of renal function change in SGLT-2 inhibitors users

According to the baseline eGFR values, all included patients were divided into three subgroups: eGFR >90 mL/min/1.73 m^2^, 60 < eGFR ≤90 mL/min/1.73 m^2^, and eGFR ≤60 mL/min/1.73 m^2^. Similar subgroupings were also conducted for other laboratory indicators, such as UACR, HbA1c, and UA as per the normal ranges in laboratory testing.

**FIGURE 2:**
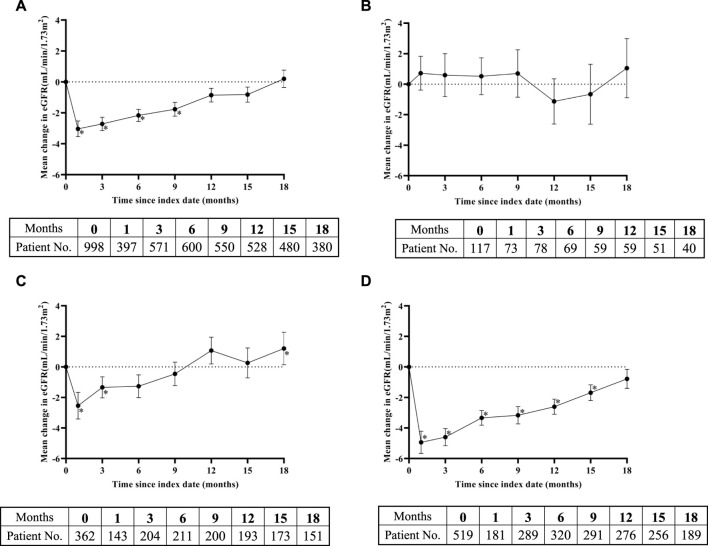
Change in eGFR levels over time after SGLT-2 inhibitors treatment: **(A)** overall study, **(B)** eGFR ≤60 mL/min/1.73 m^2^ subgroup, **(C)** 60 < eGFR ≤90 mL/min/1.73 m^2^ subgroup, and **(D)** eGFR >90 mL/min/1.73 m^2^ subgroup. Means of change in eGFR are plotted with standard error of mean. The bottom tables present the number of eGFR observations available at each time point. *, representing the *p* < 0.05 of eGFR in the time point and baseline by paired sample test.

The overall eGFR trend showed that within 15 months after the start of treatment, eGFR levels remained below the baseline level, especially in the first month after SGLT-2 inhibitors administration, when eGFR levels reached their lowest point (an average decrease of 3.04 mL/min/1.73 m^2^) (Figure 2A). However, this was followed by a stable upward trend and, hence, improvement. From the 12^th^ month onwards, the eGFR level returned to the baseline level, and no statistically significant differences were observed between eGFR and the baseline levels. However, the trend of eGFR changes was not entirely consistent in the three different subgroups. The trend of changes in the subgroup with eGFR ≤60 mL/min/1.73 m^2^ was completely different from that of the other two groups. Compared with the baseline value, the eGFR did not decrease within 9 months after taking SGLT-2 inhibitors, but slightly increased, and there was no statistical difference between the eGFR and baseline levels during the 18-month observation period (Figure 2B). The trend of changes in the subgroup with 60 < eGFR ≤90 mL/min/1.73 m^2^ was similar to the overall trend, i.e., the eGFR value significantly decreased in the first month, gradually recovered from the third month, and tended to be higher than the baseline value at the 12^th^ month. The difference between the eGFR and baseline values at the 18^th^ month reached statistical significance (Figure 2C). In the subgroup with eGFR >90 mL/min/1.73 m^2^, the trend of change was almost the same as the overall trend and approached the baseline level by the 18^th^ month (Figure 2D).

**FIGURE 3:**
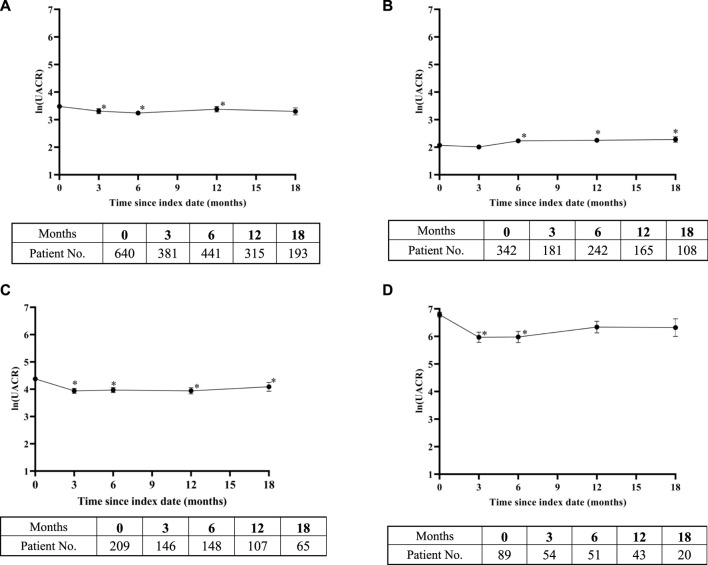
Change in ln (urinary albumin/creatinine ratio) over time after SGLT-2 inhibitors treatment: **(A)** overall study, **(B)** urinary albumin/creatinine ratio (UACR) ≤ 30 mg/g subgroup, **(C)** 30 < UACR ≤300 mg/g subgroup, and **(D)** UACR >300 mg/g subgroup. Means of change in ln (UACR) are plotted with standard error of mean. The bottom tables present the number of UACR observations available at each time point. *, representing the *p* < 0.05 of UACR in the time point and baseline by paired sample test.

In addition to eGFR, we also examined the UACR, another indicator that reflects renal function. According to the baseline UACR level, patients were divided into three subgroups: normoalbuminuria (≤30 mg/g), microalbuminuria (30–300 mg/g), and macroalbuminuria (>300 mg/g). As illustrated in Figure 3A, the natural logarithm of UACR showed minor change over the observation period. The UACR was significantly lower than the baseline UACR in the 3^rd^, 6^th^, and 12^th^ months. In the subgroup with UACR ≤30 mg/g, the UACR significantly increased from the 6^th^ month after taking SGLT-2 inhibitors (Figure 3B). After using SGLT-2 inhibitors, the UACR values of the 30 < UACR ≤300 mg/g and UACR >300 mg/g groups were lower than the baseline values (Figures 3C, D). The difference between the UACR value and baseline value in the subgroup with UACR >300 mg/g only reached statistical significance in the first 6 months.

### Changes in other indicators following SGLT-2 inhibitors administration

**FIGURE 4:**
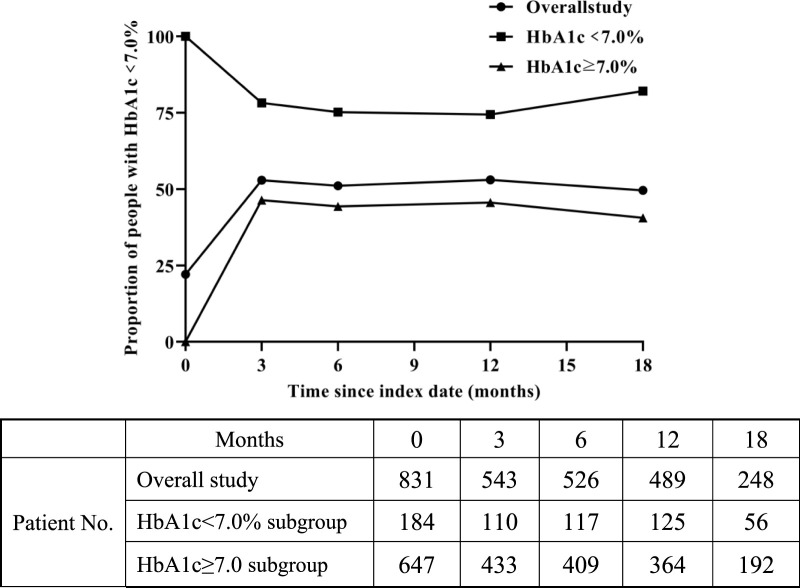
Change in proportion of people with HbA1c < 7.0% over time after SGLT-2 inhibitors treatment.

As depicted in Figure 4, the overall study results showed that the compliance rate of HbA1c reached a maximum of 53.0% in the 12^th^ month after initiation of SGLT-2 inhibitors therapy. The value fluctuated around 50% at other time points. There were 647 patients with baseline HbA1c values ≥7.0%, and the change in HbA1c compliance was similar to the overall trend. In the subgroup with baseline HbA1c < 7.0%, the HbA1c compliance rate fluctuated around 75% during the follow-up period.

After excluding patients with a history of using urate-lowering therapy drugs, we also analyzed the changes in UA values of the remaining 911 patients. We divided patients into two subgroups for analysis based on the baseline UA values. As shown in Supplemental Figure S1A, the overall UA level decreased during the observation period. The baseline mean for the subgroup UA < 357 μmol/L was 292.71 μmol/L. Although there was an increase in UA levels during the follow-up period in this subgroup, the overall average value did not exceed the limit (Supplemental Figure S1B). In the subgroup with UA ≥ 357 μmol/L, UA levels remained significantly lower than baseline throughout the entire follow-up period after initiating SGLT-2i treatment (Supplemental Figure S1C).

After excluding patients with a history of using lipid modulating drugs, we analyzed the trend of blood lipid changes in the remaining patients. The LDL-C, TG, and TC values were significantly lower than the baseline level in the first 9 months following SGLT-2 inhibitors administration (Supplemental Figures S2A–C). The TG remained significantly lower than the baseline value 12 months after SGLT-2 inhibitors administration. However, HDL-C showed an overall upward trend, but only the values in the 9^th^ month showed statistical differences from the baseline values (Supplemental Figure S2D).

## Discussion

This was the first real-world study to focus on renal protection of SGLT-2 inhibitors in patients with T2DM with different baseline renal functions, conducted in mainland China. Although the data was only from the outpatient prescription database of one hospital, Peking University People’s Hospital is one of the first hospitals in mainland China to use SGLT-2 inhibitors drugs. Moreover, adequate outpatient flow ensures population diversity, and that the results are applicable to a wider Asian population. This study further validated the protective effect of SGLT-2 inhibitors on renal function in T2DM patients based on real-world records.

Until now, only a few studies have reported the effect of SGLT-2 inhibitors on renal prognosis in patients with different baseline renal functions. The proportions of patients in different subgroups in our study were similar to that in the CVD-REAL trial, which was the first largest transnational observational cohort study to evaluate the effects of SGLT-2 inhibitors treatment on kidney prognosis of patients in the real world (Heerspink et al., 2020b). However, the baseline eGFR of patients in the CVD-REAL trial was significantly higher than that of our study. A cohort study from Taiwan, China, explored the effects of SGLT-2 inhibitors on the renal prognosis of Asian patients with T2DM in the real world (Lin et al., 2021). The average baseline eGFR level of patients included in Taiwanese study was similar to that of our study, however the population composition based on baseline eGFR grouping was significantly different. The proportion of patients with atherosclerosis cardiovascular disease reached 51.7% in our study, significantly higher than that in the former studies (Heerspink et al., 2020b; Lin et al., 2021). In addition, the 18th month follow-up rates of the three subgroups in our study were higher than those of other real-world studies (Lin et al., 2019; Heerspink et al., 2020b; Lin et al., 2021). Although there were differences in patient characteristics and study design between our study and previous studies (Heerspink et al., 2016; Heerspink et al., 2020b; Lin et al., 2021), the overall trend of eGFR changes in patients using SGLT-2 inhibitors was consistent; the decrease in eGFR reached a maximum value in the first month and then began to rise gradually and tended to return to baseline level. However, some studies have shown that SGLT-2 inhibitors have the advantage of slowing the downward trend of eGFR, but do not show an upward trend (Wheeler et al., 2021; Zannad et al., 2021; Wheeler et al., 2022). In summary, the results of our research provided the support for the improvement of renal function by SGLT-2 inhibitors in the real world.

The trend of eGFR variation in the subgroup with eGFR ≤60 mL/min/1.73 m^2^ in most studies was consistent with the overall results of the study (Chertow et al., 2021; Lin et al., 2021). However, in our study, the trend of eGFR changes during follow-up in this subgroup was significantly different from the overall results, while the results of other subgroups were similar to those of previous studies (Chertow et al., 2021; Lin et al., 2021). The average baseline eGFR in the eGFR ≤60 mL/min/1.73 m^2^ subgroup in our study was 47.57 mL/min/1.73 m^2^, and the average eGFR within 9 months after taking SGLT-2 inhibitors was higher than the baseline value, and then began to decline. Another study showed similar results, in eGFR ≤60 mL/min/1.73 m^2^ subgroup, which showed that the eGFR was greater than the baseline value within 2 years after treatment with dapagliptin, and then began to decline (Mosenzon et al., 2019). In addition, the CVD-REAL study reported that in the corresponding subgroup (n = 973), the eGFR of patients after taking SGLT-2 inhibitors increased by 0.22 mL/min/1.73 m^2^, however, the average value of eGFR in the subgroup and its change over time were not reported (Heerspink et al., 2020b). It is possible that differences in renal function among patients included in the studies led to different conclusions. Moreover, considering that not all patients had a corresponding eGFR value at each time point during the follow-up period and the number of patients included in the three subgroups was significantly different, careful interpretation of the results is required. Thus, increasing the sample size of the eGFR ≤60 mL/min/1.73 m^2^ subgroup may increase robustness in this study. In summary, our results indicated that SGLT-2 inhibitors have a certain improvement effect on renal function in T2DM patients with eGFR ≤60 mL/min/1.73 m^2^ over a certain period of time.

The differences in SGLT-2 inhibitors action on renal function may also be related to follow-up time. Some studies have shown that eGFR decreases sharply in a dose-dependent manner within a few weeks, (Heerspink et al., 2016; Lin et al., 2019), while the probability of kidney-related events increases with time (Lin et al., 2019). However, the potential mechanism of SGLT-2 inhibitors in protecting the kidneys is not fully understood. The results of the EMPA-REG trial showed that the potential change in glomerular pressure contributes to the improved renal end point (Wanner et al., 2016). Several studies report that the renal hemodynamic mechanism mediated by natriuretic and glucose-induced osmotic diuresis is the main reason for the short-term biphasic changes in renal function caused by SGLT-2 inhibitors (Heerspink et al., 2018; Cherney et al., 2020; van Bommel et al., 2020). Moreover, the type of SGLT-2 inhibitors also affects the degree of eGFR decrease in patients. Huilin et al. pointed out that empagliflozin causes fewer kidney-related events than dapagliflozin, (Tang et al., 2017), however, Lin et al. reported contradictory results (Lin et al., 2019). Thus, in future, research should focus on the correlation between SGLT-2 inhibitors type and the trend of eGFR changes.

Our study also analyzed the UACR trend over time after using SGLT-2 inhibitors in patients with T2DM. Although the sample size for UACR was significantly smaller than that of eGFR, we still performed subgroup analysis according to the baseline value. During the follow-up period, patients with baseline proteinuria showed varying degrees of improvement. For patients with microalbuminuria at baseline, the maximum reduction of UACR after taking SGLT-2 inhibitors reached 38.9%. Patients with clinical proteinuria at baseline showed the maximum decrease in UACR during the observation period, which was 47.5%. For patients with proteinuria in our study, the degree of reduction in proteinuria caused by SGLT-2 inhibitors was similar to the results of large randomized controlled trials, ranging from 18% to 40%. (Vlado et al., 2018; Perkovic et al., 2019; Jongs et al., 2021). The prespecified analysis of the DAPA-CKD trial showed that SGLT-2 inhibitors can reduce proteinuria in patients with diabetes. (Pollock et al., 2019; Heerspink et al., 2020a; Jongs et al., 2021). Our study further confirmed the role of SGLT-2 inhibitors in reducing proteinuria in patients with T2DM. Moreover, Jongs et al. found a significant reduction of UACR during dapagliflozin treatment was related to eGFR decrease during the follow-up period. (Jongs et al., 2021). In future, the sample size needs to be increased when studying the correlation between eGFR and UACR.

Moreover, we also analyzed the impact of SGLT-2 inhibitors on HbA1c levels. Most of the patients included in our study were treated with SGLT-2 inhibitors in combination with other types of hypoglycemic drugs. We conducted statistical analyses on the proportion of patients whose HbA1c reached the standard value after initiation of SGLT-2 inhibitors and found that the overall compliance rate of HbA1c showed an upward trend after initiating SGLT-2 inhibitors treatment. After the follow-up period, the overall HbA1c decreased by an average of 1.1% compared to baseline. Similarly, other studies have shown that SGLT-2 inhibitors can reduce HbA1c in patients with T2DM by 0.11%–0.9% after a treatment cycle of not less than 6 months (Jabbour et al., 2014; Ridderstråle et al., 2014; Rodbard et al., 2019; Inzucchi et al., 2021).

In addition, our results further confirmed that SGLT-2 inhibitors can reduce UA levels in patients with high UA levels (Doehner et al., 2022; Ferreira et al., 2022; Suijk et al., 2022). During the follow-up period, the time point for reaching the maximum decrease, with the value of −57.97 μmol/L, was 1 month after initiating SGLT-2 inhibitors treatment in this subgroup, which was consistent with previous studies (Doehner et al., 2022). The mechanism of SGLT-2 inhibitors in reducing UA levels has been shown to be potentially different from that of UA-lowering drugs (McDowell et al., 2022). La et al. (2022) found that the reduction of UA by SGLT-2 inhibitors was related to its inhibition of low-grade inflammation. In addition, animal experiments showed that empagliflozin attenuates hyperuricemia by upregulation of ABCG2 via the AMPK/AKT/CREB signaling pathway in type 2 diabetic mice (Lu et al., 2020). Butt et al. (2023) suggested that the lowering effect of SGLT-2 inhibitors on UA can reduce the required dosage of urate-lowering therapy drugs. Although the mechanism of lowering UA by SGLT-2 inhibitors is not yet clear, this study further confirms the urate-lowering effect of SGLT-2 inhibitors in the real world.

We also investigated the effect of SGLT-2 inhibitors on blood lipids. We analyzed the blood lipid data of the remaining patients, particularly LDL-C, after excluding those with a history of using lipid modulating drugs. Currently, reports on the role of SGLT-2 inhibitors in LDL-C have been controversial (Fadini et al., 2018; Kinguchi et al., 2019; Sezai et al., 2019; Berkovic et al., 2020; Osto et al., 2023). In our study, although there was no significant statistical difference in LDL-C level between patients at the end of the follow-up period and the baseline, LDL-C level was significantly lower than the baseline value at 1, 3, and 6 months after taking SGLT-2 inhibitors, indicating that SGLT-2 inhibitors had a certain lipid modulating effect. Sezai et al. (2019) have suggested that the improvement of LDL-C levels may be related to the reduction of fat volume in patients taking SGLT-2 inhibitors. In addition, the SGLT-2 inhibitors effect on TC was similar to that of LDL-C, and there was a trend towards lower TG after SGLT-2 inhibitors usage. However, SGLT-2 inhibitors had almost no effect on HDL-C. Therefore, SGLT-2 inhibitors can reduce blood lipids, temporarily, with the effect gradually disappearing over time. Although the mechanism and effect of SGLT-2 inhibitors on blood lipids remains controversial, our retrospective study based on the real world excluded the interference of lipid modulating drugs, which has strong persuasiveness. Previous research has shown that SGLT-2 inhibitors can reduce oxidative stress associated with T2DM, improve insulin sensitivity and lead to better cardiovascular outcomes and renal advantages (Andreadi et al., 2023). Therefore, in T2DM patients, the use of SGLT-2 inhibitors improves cardiovascular outcomes and controls metabolic consequences. People has attached more attention to the importance of SGLT-2 inhibitors in the treatment of diabetes in patients with cardiovascular diseases. Overall, our study still provides a practical basis for the protective effect of SGLT-2 inhibitors on the cardiovascular system.

This study had some limitations. First, this study was conducted in a single hospital, thus the sample size included in the study was small, especially in the eGFR ≤60 mL/min/1.73 m^2^ subgroup. Second, the relevant laboratory examinations depended on patient compliance, resulting in significant data loss during the follow-up period. In addition to the small number of patients with proteinuria or CKD, most doctors tended to examine eGFR but ignored UACR, which made it difficult to collect sufficient UACR data. Third, we did not conduct further research on the effect of SGLT-2 inhibitors type and dosage on renal function in patients. Also, a longer follow-up time is required to further study the long-term effects of SGLT-2 inhibitors on the kidneys. Fourth, we only focused on the clinical efficacy of SGLT-2 inhibitors and did not perform statistical analysis on any acute kidney injury or urinary tract infection that they may cause. Finally, no in-depth analysis was performed on some mixed factors that may affect the observation indicators.

## Conclusion

Our results indicate that during the 18-month follow-up, SGLT-2 inhibitors can improve renal function in patients with T2DM regardless of their baseline renal function. Although eGFR may decline sharply in the initial stage of medication, it gradually improves with prolonged medication. Our study further demonstrates that SGLT-2 inhibitors can reduce proteinuria in patients with T2DM. On the basis of existing clinical trials and real-world studies, our findings provide further evidence for the use of SGLT-2 inhibitors and their renal benefits in Asian populations.

## Data Availability

The raw data supporting the conclusions of this article will be made available by the authors, without undue reservation.
