# Supplementary material for: Retrospective analysis of the effect of SGLT-2 inhibitors on renal function in patients with type 2 diabetes in the real world
Source: Front Pharmacol. 2024 Aug 5;15:1376850. doi: 10.3389/fphar.2024.1376850 (PMC11330817; doi:10.3389/fphar.2024.1376850)
Supplement: Supplementary file 1 [file Table1.docx]

**Supplemental Table 1. Baseline characteristics of 998 patients including overall and subgroups stratifed by baseline** **eGFR levels (eGFR ≤ 60, 60** ~ **90, > 90 mL/min/1.73 m^2^)**

| Characteristics | Overall | eGFR≤60 ml/min/1.73m^2^ | 60<eGFR≤90ml/min/1.73m^2^ | eGFR>90ml/min/1.73m^2^ |
| --- | --- | --- | --- | --- |
|  | N=998 | N=117 | N=362 | N=519 |
| Concomitant medication, n (%) | | | | |
| Glucose-lowering drug | | | | |
| Metformin | 772(77.4) | 41(35.0) | 276(76.2) | 455(87.7) |
| Sulfonylurea | 230(23.0) | 21(17.9) | 84(23.2) | 125(24.1) |
| Glinides | 68(6.8) | 16(13.7) | 32(8.8) | 20(3.9) |
| Acarbose | 261(26.2) | 48(41.0) | 111(30.7) | 102(19.6) |
| Thiazolidinedione | 20(2.0) | 2(1.7) | 10(2.8) | 8(1.5) |
| DPP-4 inhibitors | 387(38.8) | 33(28.2) | 132(34.5) | 222(42.8) |
| GLP-1 receptor agonist | 154(15.4) | 9(7.7) | 36(9.9) | 109(21.0) |
| Insulin | 362(36.3) | 56(47.9) | 116(32.0) | 190(36.6) |
| Initial drug treatment | | | | |
| SGLT-2 inhibitors | 69(6.9) | 16(13.7) | 34(9.4) | 19(3.7) |
| Metformin+SGLT-2 inhibitors | 141(14.1) | 4(3.4) | 59(16.3) | 78(15.0) |
| Metformin+SGLT-2 inhibitors+others^a^ | 631(63.2) | 37(31.6) | 217(59.9) | 377(72.6) |
| SGLT-2 inhibitors+others^a^ | 157(15.7) | 60(51.3) | 52(14.4) | 45(8.7) |
| Other medications | | | | |
| ACE inhibitors | 80(8.0) | 12(10.2) | 37(10.2) | 31(6.0) |
| ARB | 461(46.2) | 70(59.8) | 191(52.8) | 200(38.5) |
| β-blocker | 365(36.6) | 60(51.3) | 150(41.4) | 155(29.9) |
| CCB | 312(31.3) | 53(45.3) | 136(37.6) | 123(23.7) |
| Diuretic | 33(3.3) | 10(8.5) | 15(4.1) | 8(1.5) |
| Statin | 710(71.1) | 88(75.2) | 274(75.7) | 348(67.1) |
| Antiplatelet Drugs | 193(19.3) | 34(29.1) | 84(23.2) | 75(14.4) |
| Urate-Lowering Therapy Drugs | 82(8.2) | 18(15.4) | 42(11.6) | 22(4.2) |

Abbreviations are as follows: eGFR, estimated glomerular fltration rate; DPP-4 inhibitors, dipeptidyl peptidase 4 inhibitors; GLP-1 receptor agonist, glucagon-like peptide 1 receptor agonist; SGLT-2 inhibitors, Sodium glucose cotransporter-2 inhibitors. ACE inhibitors, angiotensin-converting enzyme inhibitors; ARB, angiotensin receptor blocker; CCB, calcium channel blocker

Others^a^ : including sulfonylureas, glinides, acarbose, thiazolidinedione, DPP-4 inhibitors, GLP-1 receptor agonist and insulin.
