# Supplementary material for: Retrospective analysis of the effect of SGLT-2 inhibitors on renal function in patients with type 2 diabetes in the real world
Source: Front Pharmacol. 2024 Aug 5;15:1376850. doi: 10.3389/fphar.2024.1376850 (PMC11330817; doi:10.3389/fphar.2024.1376850)
Supplement: Supplementary file 2 [file Image2.pdf]

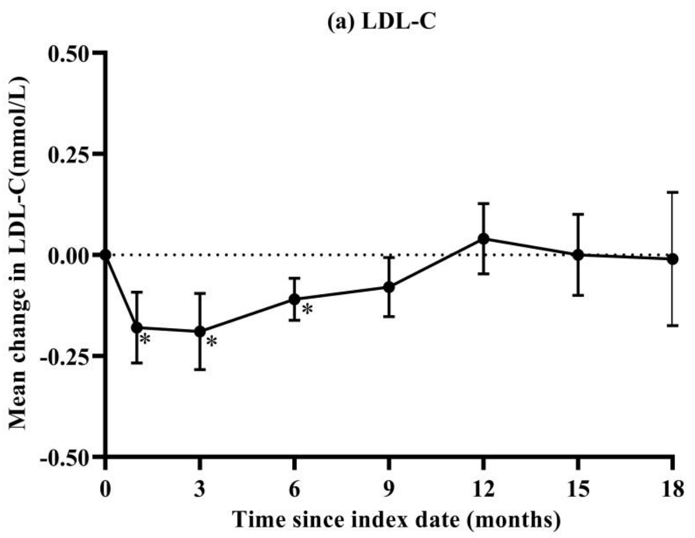

Months	0	1	3	6	9	12	15	18
Patient No.	280	75	189	156	128	86	56	24

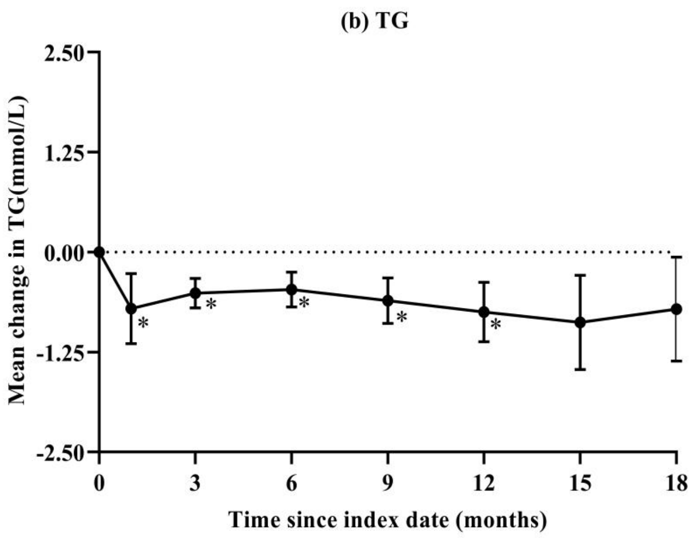

Months	0	1	3	6	9	12	15	18
Patient No.	283	75	192	158	129	87	56	22

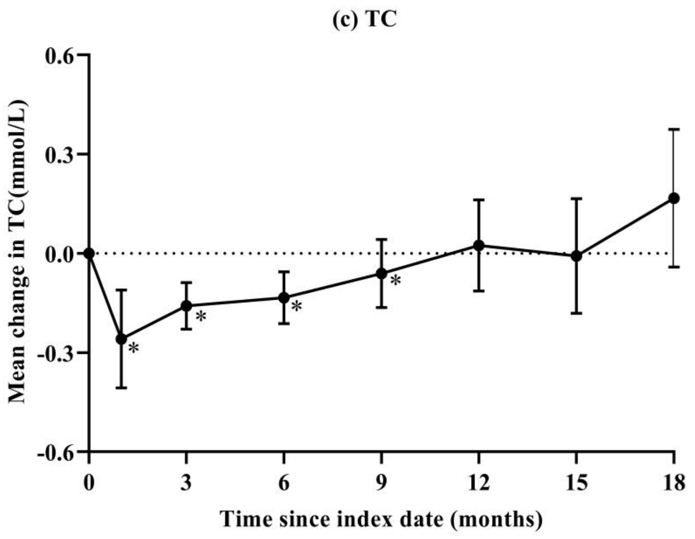

Months	0	1	3	6	9	12	15	18
Patient No.	283	75	191	158	129	87	55	22

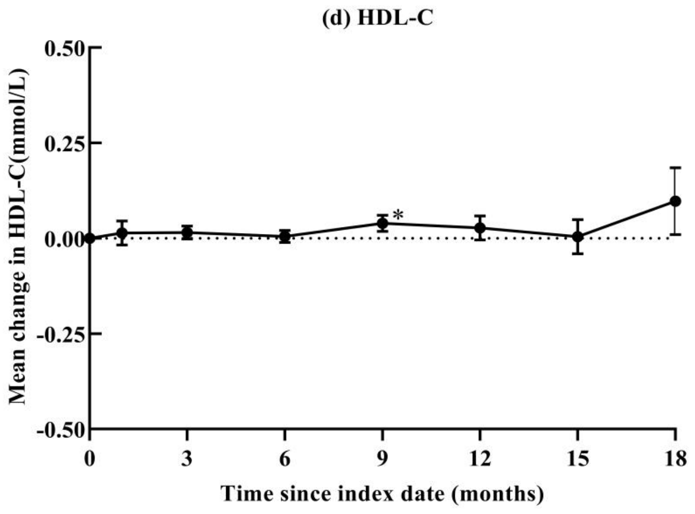

Months	0	1	3	6	9	12	15	18
Patient No.	280	75	189	154	128	86	56	21

Supplemental Figure 2. Change in blood lipid levels over time after SGLT-2 inhibitors treatment: a Low density lipoprotein cholesterol (LDL-C), b Triglyceride(TG), c Total cholesterol(TC), and d High density lipoprotein cholesterol (HDL-C). Means of change were plotted with standard error of mean. The bottom tables present the number observations available at each time point. *, representing the $p < 0.05$ in the time point and baseline by paired sample test.
