# Supplementary material for: Retrospective analysis of the effect of SGLT-2 inhibitors on renal function in patients with type 2 diabetes in the real world
Source: Front Pharmacol. 2024 Aug 5;15:1376850. doi: 10.3389/fphar.2024.1376850 (PMC11330817; doi:10.3389/fphar.2024.1376850)
Supplement: Supplementary file 3 [file Image1.pdf]

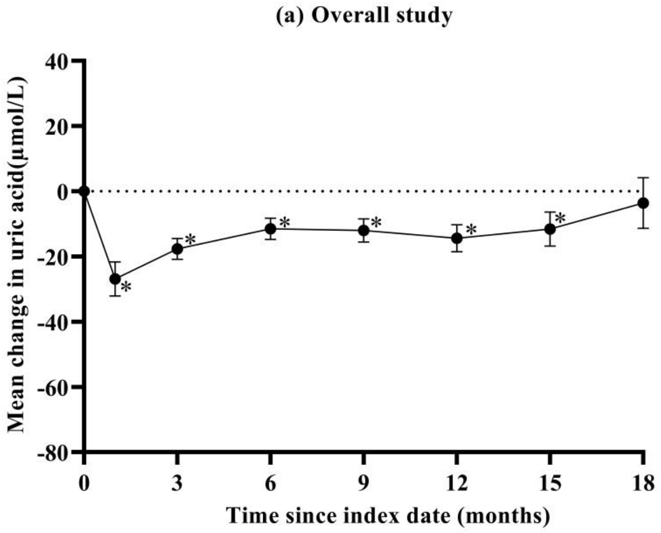


| Months | 0 | 1 | 3 | 6 | 9 | 12 | 15 | 18 |
|---|---|---|---|---|---|---|---|---|
| Patient No. | 911 | 229 | 586 | 531 | 463 | 357 | 237 | 115 |

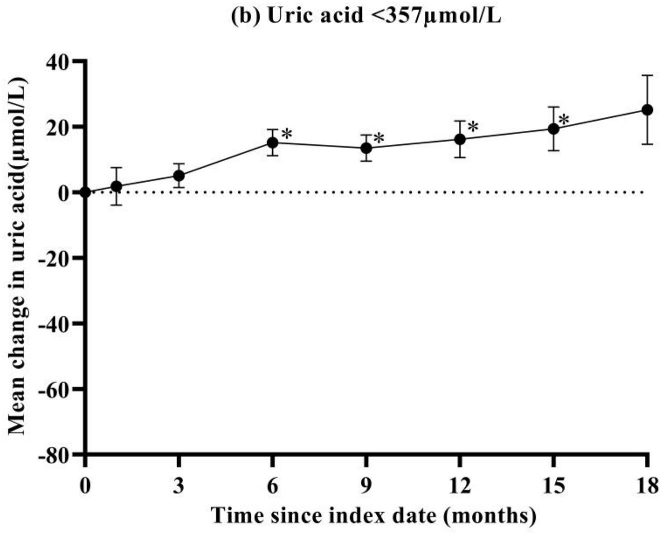


| Months | 0 | 1 | 3 | 6 | 9 | 12 | 15 | 18 |
|---|---|---|---|---|---|---|---|---|
| Patient No. | 502 | 119 | 325 | 292 | 253 | 180 | 121 | 59 |

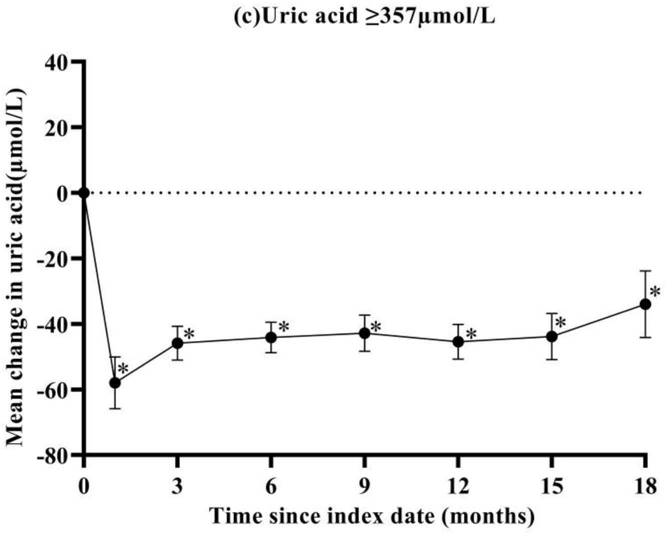


| Months | 0 | 1 | 3 | 6 | 9 | 12 | 15 | 18 |
|---|---|---|---|---|---|---|---|---|
| Patient No. | 409 | 110 | 261 | 239 | 210 | 177 | 116 | 56 |

Supplemental Figure 1. Change in uric acid (UA) levels over time after SGLT-2 inhibitors treatment: a overall study, b UA < 357μmol/L subgroup, and c UA ≥ 357μmol/L subgroup. Means of change in UA are plotted with standard error of mean. The bottom tables present the number of UA observations available at each time point. *, representing the $p < 0.05$ of UA in the time point and baseline by paired sample test.
